# C-756-02. Communication and Language Outcomes After Pediatric Burn Injury: Impact of Age and Burn Location

**DOI:** 10.1093/jbcr/irag033.046

**Published:** 2026-04-06

**Authors:** Sophia McLaughlin, Pengsheng Ni, Madeleine McGwin, Agnes McDonough, Ludwik K Branski, Tina L Palmieri, Mary D Slavin, J Michael Murphy, Jeffrey C Schneider, Lewis E Kazis, Colleen M Ryan

**Affiliations:** Shriners Children's - Boston, Boston, MA; Boston University School of Public Health, Boston, MA; Shriners Children's - Boston, Boston, MA; Shriners Children's - Boston, Boston, MA; University of Texas Medical Branch and Shriners Hospital for Children, Galveston, TX; University of California Davis Health, Shriners Children’s - Northern California, Sacramento, CA; Massachusetts General Hospital Brigham - Spaulding, Boston, MA; Massachusetts General Hospital, Boston, MA; Spaulding Rehabilitation Hospital, Mass General Brigham, Harvard Medical School, Boston, MA; Boston University School of Public Health, Boston, MA; Massachusetts General Hospital, Harvard Medical School, Boston, MA

## Abstract

**Introduction:**

Burn injuries in early childhood may have lasting impacts that extend beyond physical recovery, yet their association with developmental outcomes such as communication and language is not well understood. Early childhood is a critical period for language development, and injuries involving functionally important or visible body areas may disrupt these processes. This study examines how age and burn location influence communication and language outcomes in young children, as measured by the communication and language domain of the Preschool Life Impact Burn Recovery Evaluation for children 1–5 years of age (PS-LIBRE1–5).

**Methods:**

Data were obtained from parents/guardians of children 1 to 5 years of age with burn injuries. Responses to the calibration field-tested PS-LIBRE Profile (188 items) were measured on a scale of frequency or ability levels. Item responses were coded from 0 to 4, where higher scores reflected better functioning. Twenty items identified through confirmatory factor analysis loaded strongly onto the communication and language factor domain. Communication and language scores were generated from Rasch modeling and transformed into T-scores (mean = 50, SD = 10), where higher scores denote a higher level of functioning. Multivariable linear regression models were stratified by age group (< 48 months vs. ≥48 months) to evaluate associations between age at assessment, burns to one or more critical areas (hand, face, genitalia, or foot), and communication and language scores.

**Results:**

The mean child age was 3.0 ± 1.5 years (n = 498), and 83% of respondents were mothers. The average total body surface area burned was 4.2% ± 7.8% (SD), and the mean time since injury was 1.1 ± 1.32 years. Burns to one or more critical areas were present in 70.3% of children. Communication and language scores demonstrated a positive association with age up to 48 months, after which the relationship plateaued (Fig. 1). In children aged ≥48 months, burns to critical areas were associated with significantly lower scores (–2.75 points, *p*=.017; Fig. 2), whereas age at survey completion was not significantly associated with outcomes (*p*=.24). In contrast, among children <48 months, each additional month of age was associated with a 0.65-point increase in scores (*p*<.001), and burns to critical areas were not significantly associated with communication and language outcomes (*p*=.89).

**Conclusions:**

Communication and language development is strongly age-dependent in early childhood, but among older children, burns involving critical areas are linked to significantly poorer outcomes.

**Applicability of Research to Practice:**

Assessing communication and language is important in pediatric burn rehabilitation, as these skills are likely significantly impacted by burn injury. The PS-LIBRE1–5 provides a standardized tool for evaluating communication and language outcomes, supporting targeted rehabilitation and treatment planning for young children with burn injuries.

**Funding for the study:**

This work was supported by Foundation Funding (Grants #72000, #79136, and #79138), and in part by the National Institute on Disability, Independent Living, and Rehabilitation Research (Grant #90DPBU0008).

